# Supplementary material for: Four-dimensional entanglement distribution over 100 km
Source: Sci Rep. 2018 Jan 16;8:817. doi: 10.1038/s41598-017-19078-z (PMC5770424; doi:10.1038/s41598-017-19078-z)
Supplement: Supplementary file 1 — Supplementary Information [file 41598_2017_19078_MOESM1_ESM.pdf]

Four-dimensional entanglement distribution over 100 km: Supplementary information

Takuya Ikuta* and Hiroki Takesue

NTT Basic Research Laboratories, NTT Corporation,

3-1 Morinosato Wakamiya, Atsugi, Kanagawa 243-0198, Japan

(Dated: November 20, 2017)

* ikuta.takuya@lab.ntt.co.jp

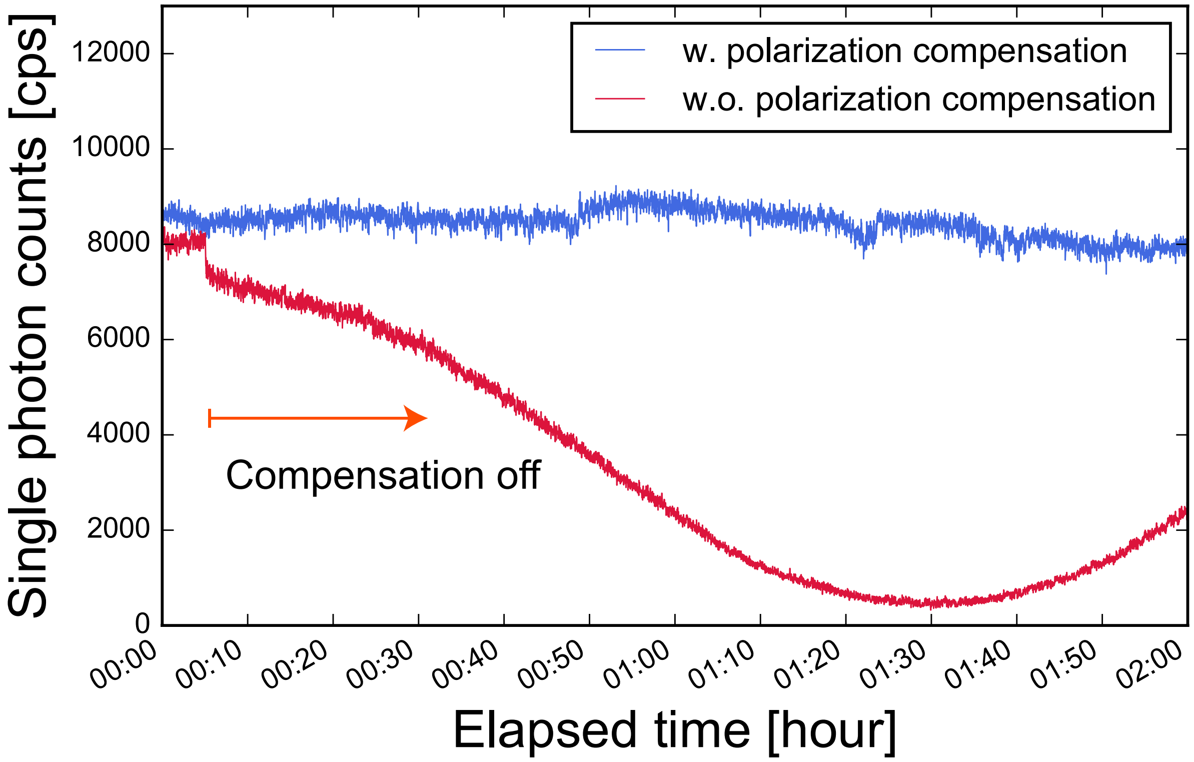

Supplementary Figure 1. Single photon counts with and without polarization compensation. The red line shows the case where the compensation was turned off after 5 minutes.

SUPPLEMENTARY NOTES

Here we describe our system for the polarization stabilization. As described in the main text, a polarizer is placed in front of the MZIs. As a result, we can compensate the polarization state by giving a feedback to the remote controllable polarization controller to maximize the single photon counts at the SNSPDs. We employed a multifunction polarization controller (MPC-201, General Photonics Co.) to stabilize the polarization state. The polarization controller contains four components to rotate the polarization. The first and third components correspond to polarization rotation of around the +45 degrees linear polarization axis of the Poincaré sphere. On the other hand, the second and fourth components correspond to polarization rotation around the horizontal linear polarization axis of the Poincaré sphere. Each polarization rotation can be controlled with a resolution of 0.01π rad. For the polarization compensation, we provided the polarization controller with

feedback as follows. The initial rotation angles of the ith component is given by ϕ_{i-1}. We swept the rotation angle of the first component from $\phi_0 - 0.1\pi$ to $\phi_0 + 0.1\pi$ and recorded a total single count at SNSPD 1 and 2 in one second for each rotation angle. Then we updated ϕ_0 to the rotation angle which showed the highest total single count.

We stabilized the polarization state by repeating this step for the second, third, and fourth components sequentially. Supplementary figure 1 shows variations of the single photon count rates at SNSPD 2 for Alice with and without the polarization compensation. The single photon count rate without it degraded to ≈ 0 counts after one-and-a-half hours due to the slow change in the fiber birefringence. On the other hand, our feedback system maintained high single count rates during the long-time measurement as seen by the blue line in supplementary figure 1. This means that we successfully compensated for the fluctuation of the polarization state in the fiber by using the simple feedback control.
